# Durability and Sustainability of Cement and Concrete Composites

**DOI:** 10.3390/ma16165693

**Published:** 2023-08-19

**Authors:** Jin Yang, Xingyang He

**Affiliations:** 1 School of Civil Engineering, Architecture and Environment, Hubei University of Technology, Wuhan 430068, China; 2 Building Waterproof Engineering and Technology Research Center of Hubei Province, Hubei University of Technology, Wuhan 430068, China

Durability and sustainability are important objectives within the development of cement and concrete composites [1], and have increasingly attracted global attention from scientists, engineers, and technologists. The durability of cement and concrete composites is of great significance for the service and safety of structures, and it also helps to reduce maintenance costs and waste of resources caused by insufficient durability in the latter stages of a structure’s life. Another important aspect is the sustainable development of cement and concrete composites in order to accomplish virtuous recycling and a healthy balance between the development of concrete technologies and resources and the environment; minimize the waste of resources used for repair and demolition and the generation of construction waste [2,3]; use a large amount of industrial solid wastes instead of high-emission cement [4]; and reduce resource and energy consumption, and environmental pollution.

This Special Issue, “Durability and Sustainability of Cement and Concrete Composites” aims to collate a set of papers presenting recent advances in the field of durability and sustainability of cement and concrete composites, including design, processing, performance, curing and maintenance, and on-site applications of cement and concrete composites. In total, we received 19 submissions, and 12 of them were published. Researchers from China, the United Kingdom, Japan, and France chose to share their recent research findings via this particular issue.

Solid wastes (such as fly ash, granulated blast-furnace slag, and phosphorus slag), as alternative cementitious binders, help to reduce carbon footprint and improve the sustainability of cement and concrete composites. Ground-granulated blast-furnace slag (GGBS) is a kind of high-activity waste slag formed via the quenching and granulation of silicate and alumino-silicate as the main components in blast-furnace smelting pig iron. Feng et al. [5] prepared an alkali-activated GGBS material with CaO and MgO as activators. It was reported that the addition of MgO and CaO helps to improve the mechanical strengths of alkali-activated GGBS. Yang et al. [6] developed a phosphorus slag (PS)-based geopolymer with wet-grinding ultrafine fly ash as an aluminate supplement. It was found that the compressive strength of the PS-based geopolymer was increased by up to 28.1%.

In order to improve the negative effects of alternative cementitious binder on the early mechanical properties of cement-based materials, Wang et al. [7] used polyaluminum sulfate as an alkali-free liquid accelerator to promote the early hydration rate of cement. It was found that its mechanical strengths were improved by consuming a large amount of Ca(OH)_2_ and forming more compact hydration products. Furthermore, Yan et al. [8] used natural minerals (calcined attapulgite and montmorillonite) as additives to improve the early and late compressive strength of cement paste.

In addition, low reactive or inert solid waste can also be used as an alternative aggregate. Recycled tyre shreds and crumbs were used by Maddalena [9] to replace fine aggregate. The durability of the prepared mortar, containing tyre crumbs at lower particle sizes, was effectively improved, with negligible shrinkage and reduced water absorption. Waste glass was used as fine aggregate by Chen et al. [10], and it was found that the replacement of waste glass sand effectively improved the gas permeation resistance of mortar. Manufactured sand is also a kind of alternative aggregate with broad application prospects. Zheng et al. [11] investigated the effects of fines on the durability of high-strength manufactured sand concrete. It was suggested that the fines content of high-strength manufactured sand concrete should be controlled within 5–15%, and the durability is the best when the fines content is 10%.

The major characteristic and advantage of sustainable development is the more efficient use of energy and the greater recycling of materials, achieved by conserving and reducing waste. Therefore, improving the durability of concrete can contribute to the sustainable development of concrete. It was reported in [9] that the freeze/thaw resistance of cement mortar was significantly improved by the addition of waste tyre particles. The chloride and sulfate impermeability of concrete are effectively improved by contents of granite manufactured sand fines lower than 15% [11]. Xu et al. [12] prepared sludge ceramsite with CO_2_ curing, and investigated its influence on the corrosion resistance of cement concrete. It was found that the freeze/thaw resistance and corrosion resistance of inner steel bars were improved by the application of CO_2_-cured sludge ceramsite. It was also found by Xu et al. [13] that the corrosion resistance of inner steel bars in magnesium phosphate cement-based reactive powder concrete can be improved by adding steel fibers at appropriate dosages. Sulfate attack is one of the common durability problems of concrete. Jiang et al. [14] investigated the coupled effect of sulfate attacks and environmental temperature. It was found that sulfate attacks were more serious at 5 °C than that at 20 °C, which is related to the greater crystallization pressure caused by the conversion from thenardite to mirabilite at lower temperatures.

Furthermore, during its service life, the shrinkage and high-temperature resistance of concrete are also important factors affecting its durability. Yang et al. [15] compared various types of shrinkage compensation agents for commercial concrete used in tunnel annular secondary lining engineering. It was found that a shrinkage-reducing agent has the optimum shrinkage reduction performance, but the strength of the concrete decreased significantly compared to when other kinds of agents were used. Chen et al. [16] investigated the effect of high temperature and confining pressure on the gas permeability of cement mortar. An increase in gas permeability was observed with an increase in temperature and heating rate. At the same time, the test confining pressure will also affect gas permeability results.

The findings presented in this editorial contribute to recent progress in the durability and sustainability of cement and concrete composites. Durability and sustainability will always be the focus of cement concrete research. Therefore, low carbon emissions and long service life should be the key objectives of the future development of the concrete industry.
